# IL-17 Induction by ArtinM is Due to Stimulation of IL-23 and IL-1 Release and/or Interaction with CD3 in CD4^+^ T Cells

**DOI:** 10.1371/journal.pone.0149721

**Published:** 2016-02-22

**Authors:** Thiago Aparecido da Silva, Vania Sammartino Mariano, Aline Sardinha-Silva, Maria Aparecida de Souza, Tiago Wilson Patriarca Mineo, Maria Cristina Roque-Barreira

**Affiliations:** 1 Departamento de Biologia Celular e Molecular e Bioagentes Patogênicos, Faculdade de Medicina de Ribeirão Preto, Universidade de São Paulo, Ribeirão Preto, SP, Brasil; 2 Instituto de Ciências Biomédicas, Universidade Federal de Uberlândia, Uberlândia, MG, Brasil; University of Nebraska Medical center, UNITED STATES

## Abstract

ArtinM is a D-mannose-binding lectin extracted from the seeds of *Artocarpus heterophyllus* that interacts with TLR2 N-glycans and activates antigen-presenting cells (APCs), as manifested by IL-12 production. *In vivo* ArtinM administration induces Th1 immunity and confers protection against infection with several intracellular pathogens. In the murine model of *Candida albicans* infection, it was verified that, in addition to Th1, ArtinM induces Th17 immunity manifested by high IL-17 levels in the treated animals. Herein, we investigated the mechanisms accounting for the ArtinM-induced IL-17 production. We found that ArtinM stimulates the IL-17 production by spleen cells in BALB/c or C57BL/6 mice, a response that was significantly reduced in the absence of IL-23, MyD88, or IL-1R. Furthermore, we showed that ArtinM directly induced the IL-23 mRNA expression and the IL-1 production by macrophages. Consistently, in cell suspensions depleted of macrophages, the IL-17 production stimulated by ArtinM was reduced by 53% and the exogenous IL-23 acted synergistically with ArtinM in promoting IL-17 production by spleen cell suspensions. We verified that the absence of IL-23, IL-1R, or MyD88 inhibited, but did not block, the IL-17 production by ArtinM-stimulated spleen cells. Therefore, we investigated whether ArtinM exerts a direct effect on CD4^+^ T cells in promoting IL-17 production. Indeed, spleen cell suspensions depleted of CD4^+^ T cells responded to ArtinM with very low levels of IL-17 release. Likewise, isolated CD4^+^ T cells under ArtinM stimulus augmented the expression of TGF-β mRNA and released high levels of IL-17. Considering the observed synergism between IL-23 and ArtinM, we used cells from IL-23 KO mice to assess the direct effect of lectin on CD4^+^ T cells. We verified that ArtinM increased the IL-17 production significantly, a response that was inhibited when the CD4^+^ T cells were pre-incubated with anti-CD3 antibody. In conclusion, ArtinM stimulates the production of IL-17 by CD4^+^ T cells in two major ways: (I) through the induction of IL-23 and IL-1 by APCs and (II) through the direct interaction with CD3 on the CD4^+^ T cells. This study contributes to elucidation of mechanisms accounting for the property of ArtinM in inducing Th17 immunity and opens new perspectives in designing strategies for modulating immunity by using carbohydrate recognition agents.

## Introduction

The IL-17 family of cytokines (IL-17B, IL-17C, IL-17D, IL-17E, IL-17F) has been associated with a distinct lineage of CD4^+^ T helper (Th) lymphocytes known as Th17 cells [1, 2] that are characterized by the production of IL-17A (also named IL-17), IL-17F, and IL-22 [3]. The transforming growth factor beta (TGF-β) and the proinflammatory cytokine IL-6 are required in the initiation of Th17-cell development in mice [4, 5], whereas IL-23 is committed to expanding Th17-cell populations [6]. In addition, Th17 cells generated in the presence of IL-6, IL-1β, and IL-23, seem to have higher pathogenic potential instead of TGF-β signaling [7]. Moreover, recent studies show that MyD88 plays a role in Th17 cells by delivering IL-1 signaling during the early differentiation stage [8]. The IL-17 production, sourced by cells of both innate (including γδT cells, NK cells, neutrophils) and adaptive (Th17) systems [9], is maintained during immune and inflammatory responses by IL-23, which originates from dendritic cells and macrophages [4]. The inflammatory capacity of innate cells is enhanced by IL-17 [10] by recruiting additional inflammatory leukocytes, which eliminate microbial infections [11]. These observations were obtained in studies on the host defense against *Candida albicans* (*C*. *albicans*) [12], which demonstrated the role of Toll-Like Receptor-2 (TLR2)/Dectin-1 pathway in the production of IL-17 during the fungal infection [13].

ArtinM, a D-mannose-binding lectin, obtained from the seeds of *Artocarpus heterophyllus*, is organized as a homotetramer [14]. Each 16 kDa chain contains a carbohydrate recognition domain (CRD) with affinity to Manα1–3[Manα1–6]Man, which corresponds to the core of N-linked oligosaccharides. ArtinM induces the production of proinflammatory cytokines, triggered by the lectin interaction with glycotargets on the surface of immune cells, such as neutrophils [14–16], macrophages, dendritic cells [17], and mast cells [18]. ArtinM interaction with TLR2 N-glycans on antigen-presenting cells (APCs) induces IL-12 production [19–21]. This response is implicated in the immunomodulatory activity toward a Th1 phenotype exerted by the *in vivo* administration of ArtinM. It confers protection against infections with *Paracoccidioides brasiliensis* [17, 22], *Leishmania major* [19], *Leishmania amazonensis* [20], and *Neospora caninum* [23]. In addition, the ArtinM administration to mice infected with *C*. *albicans* is followed by the development of Th17 immunity, which contributes to confer protection against the fungal disease [24].

Our group showed that ArtinM, besides activating cells of innate immunity, acts also on murine CD4^+^ T cells, providing a direct mechanism of inducing Th1 response [25]. We hypothesized that IL-17 production verified in *C*. *albicans* infected mice could be stimulated by the proinflammatory cytokines induced by ArtinM and by the direct interaction of ArtinM with CD4^+^ T cells. In the present study, we showed that ArtinM promotes IL-23 and IL-1 release, a mechanism that contributes for the high IL-17 production. Moreover, ArtinM acts directly on CD4^+^ T cells, which release IL-17 in response to the lectin stimulus, even when the stimulus of IL-23 or IL-1 has not occurred. Our findings reveal novel mechanisms through which ArtinM exerts its immunomodulatory properties and confers protection against fungal infection.

## Materials and Methods

### Ethics statement

The Committee of Ethics in Animal Research of the College of Medicine of Ribeirão Preto at the University of São Paulo approved the animal studies, which were conducted in accordance with the Ethical Principles in Animal Research adopted by the Brazilian College of Animal Experimentation, Protocol no. 082/2012.

### Animals

Male BALB/c, C57BL/6 (wild-type, WT), MHC-II KO, IL-4 KO, IL-6 KO, IL-10 KO, IL-22 KO, IL-23 KO, IL-33R KO, IL-17R KO, MyD88 KO, IL-1R KO, TLR2 KO, TLR4 KO, CD14 KO and Dectin-1 KO mice at 6–8 weeks of age were used in this study. These mice were acquired from the animal house of the Campus of Ribeirão Preto, University of São Paulo, Ribeirão Preto, São Paulo, Brazil, and Dectin-1 KO mice were acquired from the Animal Experimentation Laboratory, Institute of Biomedical Sciences, Federal University of Uberlândia, Minas Gerais, Brazil. Animals were housed in the animal facility of the Molecular and Cellular Biology Department of the Faculty of Medicine of Ribeirão Preto, University of São Paulo, under optimized hygienic conditions.

### Lectins

ArtinM and Jacalin were purified as previously described [14] from the saline extract of *Artocarpus heterophyllus* (jackfruit) seeds via affinity chromatography on sugar columns. The lectins *Canavalia ensiformis* (ConA), *Phaseolus vulgaris* erythroagglutinin (E-PHA), *Phaseolus vulgaris* leukoagglutinin (L-PHA), *Sambucus nigra* agglutinin (SNA), *Maackia amurensis* leukoagglutinin (MAL), and *Ulex europaeus* agglutinin (UEA) were purchased from Sigma Chemical (Sigma-Aldrich, St. Louis, MO, USA). Before use, preparations of lectins were incubated for 30 min at 37°C with polymyxin B solution (50 μg/mL) (Sigma-Aldrich) to neutralize any virtual contamination with endotoxin.

### Suspensions of spleen cells and isolated CD4^+^ T cells

Mice spleens were removed aseptically, transferred to a petri dish and dissociated in a 40-μm nylon cell strainer (BD Biosciences, San Diego, CA, USA) containing Roswell Park Memorial Institute (RPMI) 1640 medium (Life Technologies, Grand Island, NY, USA). Spleen cells were centrifuged at 300 × *g* (10 min at 4°C) to yield a pellet, and the red blood cells were depleted by incubating with lysis buffer (9 parts 0.16 M ammonium chloride and one part 0.17 M Tris–HCl, pH 7.5) for 4 min at 4°C. Afterwards, the spleen cells were washed twice in RPMI 1640 and 10% fetal cow serum (FCS—Life Technologies, Grand Island, NY, USA) and centrifuged at 300 × *g* for 10 min at 4°C. The number of suspended cells was determined in a Neubauer chamber, and their viability was determined using the trypan blue exclusion method. The viability of the spleen cells was higher than 90%. This cell suspension was used to isolate CD4^+^ T cells using CD4^+^ T cell isolation kit II from Miltenyi Biotec (Auburn, CA, USA) according to the manufacturer’s instructions. To assess purity, the cells were stained with anti-CD3 PE and anti-CD4 FITC antibodies (BD Biosciences) and analyzed by flow cytometry (Guava easyCyte, Guava Technologies, Millipore, Hayward, CA, USA). Purity grades of 93–95% were achieved.

### IL-17A measurement in spleen cell supernatants

Spleen cells (2×10^6^/mL) from all mice strains were cultured in 96-well microplates in the presence of ArtinM, ConA, E-PHA, L-PHA, SNA, MAL, UEA, or Jacalin, all used in final concentrations of 0.625, 1.25, or 2.5 μg/mL. Phorbol myristate acetate (PMA; Sigma-Aldrich), ionomycin (Sigma-Aldrich), lipopolysaccharide (LPS; LPS-EB ultrapure; InvivoGen, San Diego, CA, USA), and synthetic triacylated lipopeptide (P3C4; Pam3CSK4; InvivoGen) were used as activators of spleen cells, at concentrations specified in the figure legends. After the incubation period (specified for each experiment), the spleen cells were centrifuged (300 × *g*, 10 min at room temperature), and the supernatants were collected to measure IL-17A levels.

### Lectins binding on spleen cells

The spleen cell suspension (2 × 10^6^/mL) was fixed (3% formaldehyde in phosphate-buffered saline (PBS)) for 30 min at room temperature and incubated with 1% glycine in PBS. After two washes with PBS, 25 μg/mL of either biotinylated lectin (ArtinM, ConA, E-PHA, L-PHA, SNA, MAL, UEA, or Jacalin) was added to samples of spleen cell suspensions and incubated for 45 min. After washing, the bound biotinylated lectin on spleen cells was revealed by reacting with streptavidin-fluorescein isothiocyanate (strp/FITC; 5 μg/mL; Invitrogen, Life Technologies, Camarillo, CA, USA) for 40 min. Fluorescence staining was analyzed by flow cytometry (Guava easyCyte). The percentage of stained spleen cells was determined and the level of binding of each lectin was estimated by the mean fluorescence intensity (MFI).

### Preparation of splenic adherent cells, bone marrow-derived macrophages (BMDMs), and peritoneal macrophages

Single cell suspensions of spleen cells from C57BL/6 mice were prepared as described above, adjusted to a concentration of 1 × 10^7^/ml and cultured in 24-well microplates for 24 h at 37°C in fresh medium alone. Cells in suspension were discarded by washing twice in fresh medium and the adherent cells were incubated for 7 h with ArtinM (1.25 μg/mL), LPS (1 μg/mL), or medium alone. Afterwards, the splenic adherent cells were incubated with TRIzol reagent (Life Technologies, Carlsbad, CA, USA), according to the manufacturer’s instruction, before subjecting to real-time quantitative PCR.

BMDMs were prepared from C57BL/6 mice femurs and tibias after flushing with RPMI medium to release the bone marrow cells. These cells were cultured in 100 mm × 20 mm suspension culture dish (Corning, Corning, NY, USA) containing 10 mL of RPMI 1640 medium supplemented with 20% FCS and 30% of supernatant from L929 cell cultures (LCCM, L929-cell conditioned medium). On day 4, 10 mL of RPMI medium supplemented with 20% FCS and 30% of supernatant from L929 cell cultures were added. On day 7, the non-adherent cells were removed and the dishes were washed with PBS. Then, the adherent cells (majority macrophages) were removed by applying thermal shock with cold PBS for 10 min, and washed twice with PBS. The cell concentration was determined in a Neubauer chamber, and BMDMs (1 × 10^6^/mL) were cultured in 24-well microplates for 7 h at 37°C in the presence of ArtinM (1.25 μg/mL), LPS (1 μg/mL) or medium alone. BMDMs were incubated with TRIzol reagent before subjecting to real-time quantitative PCR.

Peritoneal macrophages were obtained from the cavity of WT, TLR2 KO, CD14 KO and Dectin-1 KO mice, which were injected 3 days before with 1 mL of sterile thioglycollate medium (3%). Cells were harvested by peritoneal lavage with PBS and centrifuged at 300 × *g* (10 min at 4°C). The pellet was washed twice in RPMI 1640 with 10% FCS (300 × *g*, 10 min at 4°C). The cell concentration was determined in a Neubauer chamber, and peritoneal macrophages (2 × 10^6^/mL) were cultured for 7 h, for real-time quantitative PCR analysis, or 48 h, to determine the IL-1α, IL-1β, IL-6 and IL-12p40 levels, in the presence of ArtinM (1.25 μg/mL), LPS (1 μg/mL) or LPS plus IFN-γ (1 μg/mL + 2 ng/mL), or medium alone.

### Quantitative reverse transcription (qRT)-PCR

Total RNA was isolated from splenic adherent cells, BMDMs, peritoneal macrophages or CD4^+^ T cells using TRIzol Reagent, according to the manufacturer’s instructions. Reverse transcription of RNA into cDNA was done by the ImProm-II Reverse Transcription System (Promega, Fitchburg, WI) using oligo(dT). qRT-PCR was performed in 15 μL reactions using SYBR Green (Applied Biosystems/Life Technologies, Carlsbad, CA, USA). qRT-PCR was performed with the 7500 Real-Time PCR System (Applied Biosystems) using the following conditions: 50°C for 2 min, 95°C for 10 min, and 40 cycles of 95°C for 15 sec/60°C for 1 min. Gene expression was quantified using the ΔΔCt method and normalized to β-actin expression. PCR primers utilized were: β-actin (F: 5'AGCTGCGTTTTACACCCTTT3' / R: 5'AAGCCATGCCAATGTTGTCT3'); TGF-β (F: 5'GACTCTCCACCTGCAAGACC3' / R: 5'GGGACTGGCGAGCCTTAGTT3'); IL-23 (F: 5'TCCGTTCCAAGATCCTTCGA3' / R: 5'TGTTGGCACTAAGGGCTCAG3'); Dectin-1 (F: 5'GTGCTGGGTGCCCTAGCAT3' / R: 5'TCTGTGGGCTTGTGGTTCTCTT3').

### Depletion of macrophages, B-, Tγδ-, and CD4^+^ T-cells of the spleen cell suspension

Spleen cell suspensions, obtained as described above, were depleted of macrophages, CD4^+^ T or B cells, respectively, by using the CD11b^+^, CD4^+^ T, or B cell isolation kits (Miltenyi Biotec), according to the manufacturer’s instructions. Depletion of Tγδ cells was then accomplished by cell sorting. Briefly, spleen cell suspension was stained with biotin-conjugated anti-TCRγδ (20 μg/mL; BD Biosciences) for 40 min at 4°C, washed with PBS, and incubated with streptavidin-fluorescein isothiocyanate (strp/FITC; 10 μg/mL; Invitrogen) for 30 min at 4°C. These cells were washed twice with RPMI 1640 with 10% FCS (300 × *g*, 10 min at 4°C) and adjusted to a concentration of 1 × 10^7^/mL and then sorted using FACSAria SORP (Becton Dickinson San Jose, CA, USA) cell sorter. The depleted and non-depleted cell suspensions were plated (2 × 10^6^/mL) onto 96-well microplates and incubated at 37°C with ArtinM (1.25 μg/mL) for 48 h. The levels of IL-17 in the supernatants were determined by enzyme-linked immunosorbent assay (ELISA). The IL-17 production by the depleted cell suspension was compared to whole spleen cell suspension, and the values were expressed in percentage of inhibition of IL-17 production. In addition, we assessed and compared the IL-17 production by ArtinM stimulated spleen cell suspension obtained from MHC-II KO mice (CD4^+^ T cell-deficient) with that by ArtinM stimulated cells from WT mice.

### Stimulation of isolated CD4^+^ T cells with ArtinM

CD4^+^ T cells (2 × 10^6^/mL) obtained from WT, IL-1R KO and IL-23 KO mice were stimulated with ArtinM (1.25 μg/mL) for 48 h, after which the culture supernatants were harvested and the IL-17 levels were assessed by ELISA. In addition, CD4^+^ T cells were incubated with TRIzol reagent and used to determine the TGF-β mRNA levels by real-time quantitative PCR. A mixture of IL-1β/IL-6/IL-23 (PeproTech, Rock Hill, NJ, USA) was used, in concentrations as indicated in the figure legends, as the positive control.

### Functional competition assay between anti-CD3 antibody and ArtinM

Murine spleen cells (2 × 10^6^/mL) and purified CD4^+^ T cells (2 × 10^6^/mL) were incubated with anti-CD3 antibody (17A2 clone; 15 μg/mL; eBioscience, San Diego, CA, USA) or IgG Isotype control (A19-3 clone; 15 μg/mL; BD Bioscience), for 40 min at 4°C. After washing, the cells were cultured in 96-well microplates for 48 h at 37°C under stimulus with ArtinM (1.25 μg/mL), and IL-1β/IL-6/IL-23 (PeproTech, Rock Hill, NJ, USA) in concentrations as indicated in the figure legends. The unstimulated cells, incubated with anti-CD3 antibody or IgG Isotype control, were used as controls. ELISA was used for the measurement of IL-17A levels in the culture supernatants.

### Quantification of cytokines by ELISA

The IL-17 production was determined by ELISA Ready-SET-Go Kit (eBioscience) according to the manufacturer’s instructions. IL-1α, IL-1β, IL-6 and IL-12p40 levels in the cell culture supernatants were measured by OptEIA ELISA Kit (BD Biosciences) according to the manufacturer’s instructions.

### Statistical analysis

All data were analyzed using Prism (Graph Pad Software), and the results are presented as the mean ± standard error of the mean (SEM). Statistical determinations of the difference between means of groups were performed with analysis of variance (1-way) followed by Bonferroni’s multiple comparison tests. Differences with p < 0.05 were considered statistically significant.

## Results

### ArtinM is a potent inducer of IL-17 production by murine spleen cells

**Fig 1:**
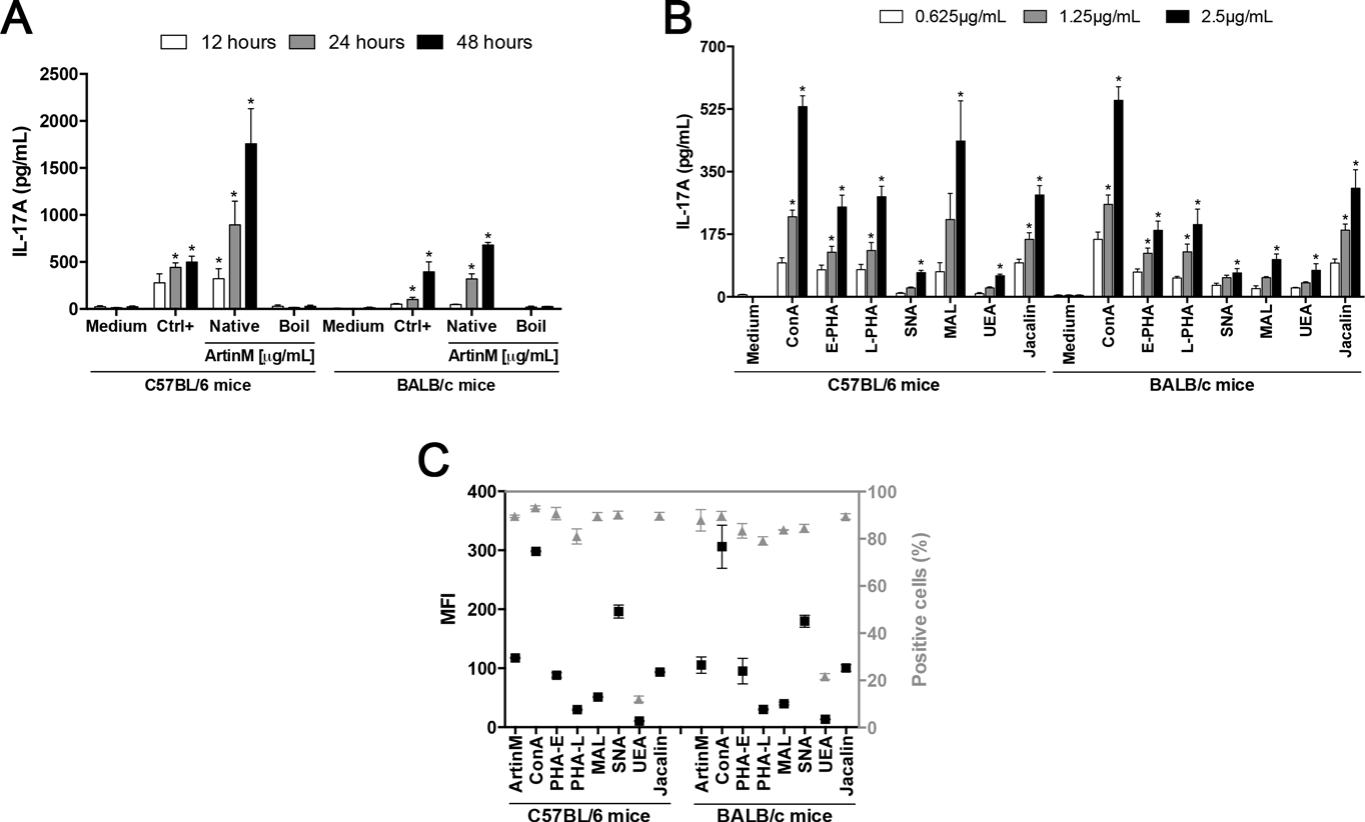
The effect of ArtinM on IL-17 production by murine spleen cells. (**A**) Spleen cell suspensions (2 × 10^6^/mL) from C57BL/6 or BALB/c mice were incubated at 37°C for 12, 24 and 48 h with boiled (Boil) or native (not boiled) samples of ArtinM (1.25 μg/mL). (**B**) Spleen cell suspensions (2 × 10^6^/mL) from C57BL/6 or BALB/c mice were incubated at 37°C for 48 h with *Canavalia ensiformis* (ConA), *Phaseolus vulgaris* erythroagglutinin (E-PHA), *Phaseolus vulgaris* leukoagglutinin (L-PHA), *Sambucus nigra* agglutinin (SNA), *Maackia amurensis* leukoagglutinin (MAL), *Ulex europaeus* agglutinin (UEA), or Jacalin in concentrations of 0.625, 1.25 or 2.5 μg/mL. In both cases (**A** and **B**), positive and negative controls were incubated with PMA plus ionomycin (81 nM + 5 μM; Ctrl+) and medium, respectively. The IL-17A concentration in the culture supernatants was measured by ELISA and compared to the levels detected in the supernatant of medium. (**C**) Fixed spleen cells from C57BL/6 or BALB/c mice were incubated with 25 μg/mL biotinylated lectins (ArtinM, ConA, E-PHA, L-PHA, SNA, MAL, UEA, or Jacalin). Lectin binding was revealed by reaction with strp/FITC (5 μg/mL), and the fluorescence was measured by flow cytometry. The median fluorescence intensity (MFI; left Y axis) and the percentage of fluorescent cells (Positive cells—%; right Y axis) were determined for each lectin. (**A**, **B,** and **C**) The results are expressed as mean ± SEM. *Significant differences with p < 0.05 in relation to Ctrl-.

We have reported previously TLR2-dependent mechanisms accounting for the ArtinM action on innate immune cells, which culminate in the induction of Th1 protective response against several intracellular pathogens. Nevertheless, the mechanism accounting for the Th17 response induced by ArtinM administration in *C*. *albicans-*infected mice [24] is still unknown. To elucidate this issue, we investigated the effect of ArtinM stimulus on IL-17 production by spleen cells from BALB/c or C57BL/6 mice. Firstly, the mixture of PMA plus ionomycin, frequently used as spleen cell activator, was assayed in terms of optimal concentrations of its components to promote IL-17 production. As per the results shown in S1 Fig, 81.0 nM PMA plus 5.0 μM ionomycin was chosen as the positive control of stimulation in the subsequent experiments. Taking into consideration that ArtinM acts as a TLR agonist, we assayed conventional agents, such as LPS and P3C4, but they did not stimulate IL-17 release (S1A Fig). Otherwise, ArtinM induced IL-17 production in a dose-dependent manner (S1B Fig). Fig 1 shows that ArtinM-stimulated spleen cells from C57BL/6 mice released the highest IL-17 concentrations, especially at 24 and 48 h incubation, when the levels were two to three times superior to that by the positive control. Spleen cells from C57BL/6 mice produced higher IL-17 levels than cells from BALB/c mice, an effect that was particularly notable after 24 and 48 h stimulation with ArtinM (Fig 1A). A boiled sample of ArtinM did not stimulate spleen cells to produce IL-17 (Fig 1A).

We have previously demonstrated that ArtinM activates spleen cells in a sugar recognition-dependent manner [25]. Then we evaluated whether other lectins with distinct sugar-binding specificities could also induce IL-17 production by spleen cells. The lectins ConA (α-Manα1-2Man); E-PHA (Galβ1-4GlcNAcβ1-2Manα1–6[GlcNAcβ1–4]Man); L-PHA (Galβ1-4GlcNAcβ1–6[Galβ1-4GlcNAcβ1–2]Man); MAL (Neu5Acα1-3Galβ1-4GlcNAc); SNA (Neu5Acα1-6Gal); Jacalin (Galβ1-3GalNAc); and UEA (Fucα1–3) were assayed at 0.625, 1.25, and 2.5 μg/mL concentrations, for 48 h. The IL-17 production was significantly promoted by all lectins in a dose-dependent manner (Fig 1B). Remarkably, none of the lectins showed an IL-17 production as high as ArtinM. Interestingly, the responses to the majority of assayed lectins (MAL was an exception) did not vary from C57BL/6 to BALB/c spleen cells, as in the case of ArtinM. These results suggest that ArtinM is a potent inducer of IL-17 production by murine spleen cells, especially by those from C57BL/6 mice.

Because the induced level of IL-17 production varied among the assayed lectins, we evaluated if it could be related to the intensity of lectin binding to the surface of spleen cells. This was assessed by the cytometric detection of biotinylated lectins (25 μg/mL) binding on the surface of spleen cell suspensions (2 × 10^6^/mL), obtained from BALB/c or C57BL/6 mice. Following reaction with strp/FITC, the proportion of fluorescent cells was greater than 80% for each of the assayed lectins, with the exception of UEA that labeled only 12% of the spleen cells (Fig 1C, right Y axis). However, the number of interactions established on the cells surface, as determined by mean fluorescence intensity (MFI) varied a lot from lectin to lectin (Fig 1C, left Y axis). Nonetheless, the observed variation did not correlate with the levels of IL-17 production induced by the lectins.

### IL-23 expression in macrophages contributes to the IL-17 production by ArtinM- stimulated spleen cells

**Fig 2:**
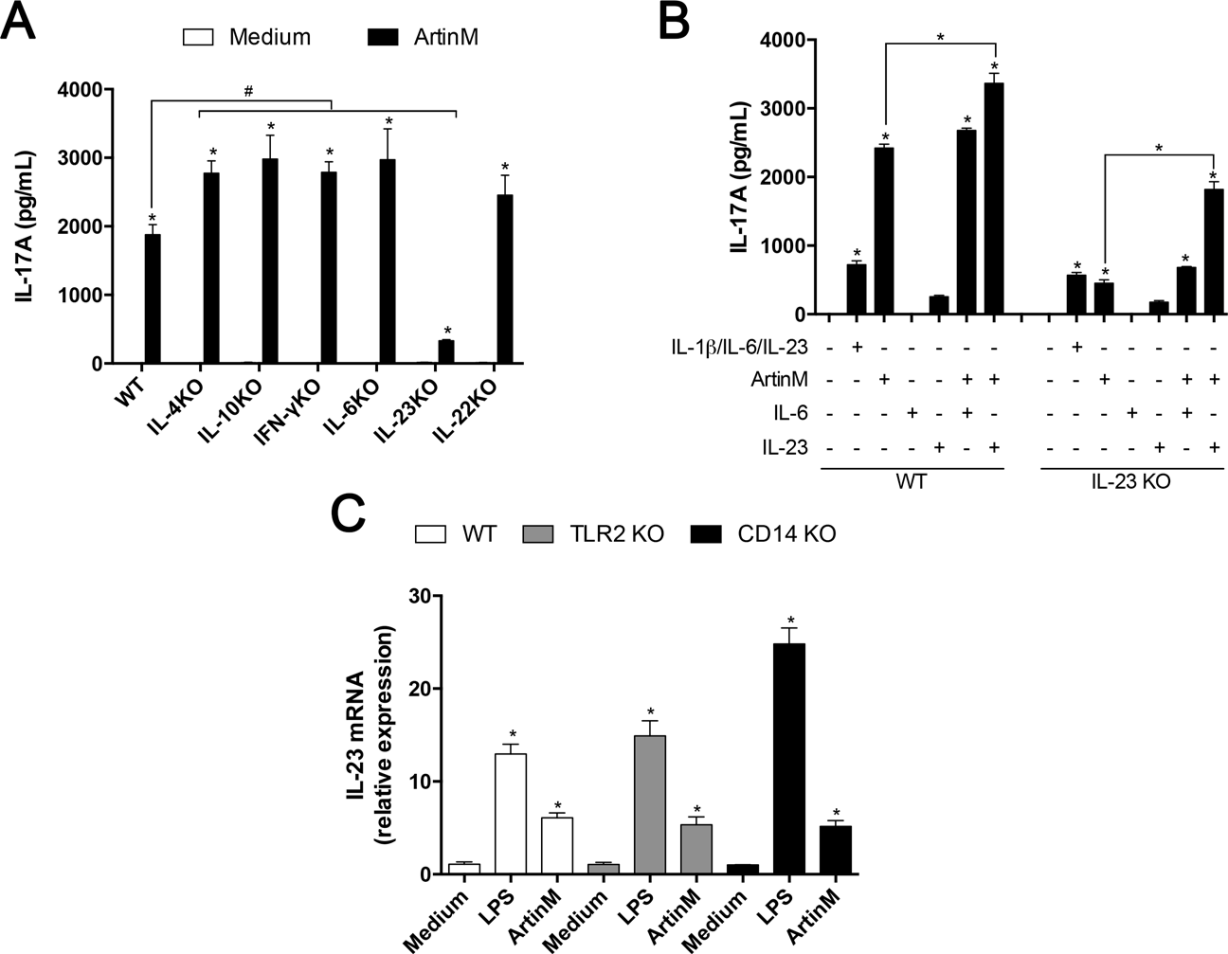
The IL-23 effect on the IL-17 production by spleen cells under ArtinM stimulus. (**A and B**) Murine spleen cells (2 × 10^6^/mL) from C57BL/6 (WT), IL-4 KO, IL-10 KO, IFN-γ KO, IL-6 KO, IL-23 KO, and IL-22 KO mice were incubated for 48 h at 37°C with ArtinM (1.25 μg/mL) or medium alone as an additional negative control. (**B**) Spleen cells from WT and IL-23 KO mice were stimulated for 48 h with ArtinM alone or in association with IL-6 or IL-23. As controls, IL-6 and IL-23 were used alone, and these cytokines were associated with IL-1β as positive control. In all cases, cytokine concentration was 10 ng/mL. (**C**) Peritoneal macrophages (2 × 10^6^/mL) from C57BL/6, TLR2 KO and CD14 KO mice were incubated with ArtinM (1.25 μg/mL) for 7 h and then the extracted RNA was used for real-time quantitative PCR of IL-23 mRNA, as described in Materials and Methods. Medium and LPS (1 μg/mL) were used as negative and positive controls, respectively. The results are expressed as the relative expression of IL-23 normalized to β-actin expression. (**A—C**) The results are expressed as mean ± SEM, and the levels of IL-17 and the expression of IL-23 were compared to that of the unstimulated cells (Medium); and additional comparison was established between the ArtinM stimulus in cells of WT and KO mice (**A**) and between the ArtinM stimulus and ArtinM plus IL-23 (**B**). Differences were considered significant when p < 0.05 (*).

Since the microenvironment afforded by a spleen cell culture includes variable concentrations of cytokines, produced by a number of cell populations, we hypothesized that the increased levels of IL-17 could result from an indirect mechanism, attributed to the ArtinM effect on several cytokines-producing cell types. We assessed this hypothesis by culturing spleen cells obtained from knockout (KO) mice for IL-4, IL-10, IFN-γ, IL-6, IL-23, or IL-22 to investigate the capacity of Th1 and Th2 cells signals (IFN-γ, IL-4, and IL-10) to inhibit IL-17 production [3]. The contribution of regulators of IL-17 production (IL-6, IL-22, and IL-23) for the maintenance of IL-17 levels induced by ArtinM was also assessed. We confirmed that the spleen cells of WT mice incubated with ArtinM increased their IL-17 production. Spleen cells from mice of all KO strain reproduced this result. The only exception was the spleen cells from IL-23 KO mice, which responded to ArtinM stimulus by producing IL-17 levels that were about six fold lower than that verified in cells from WT mice (Fig 2A). Exogenous IL-23 (10 ng/mL) enhanced the IL-17 production induced by ArtinM on spleen cells from WT and IL-23 KO mice, demonstrating a synergic effect of ArtinM and IL-23. Otherwise, exogenous IL-6 (10 ng/mL) had no effect in the IL-17 production promoted by ArtinM (Fig 2B).

After verifying that the ArtinM effect on IL-17 production by spleen cells requires IL-23, we analyzed the cytokine expression by splenic adherent cells and BMDMs in response to ArtinM stimulus. The expression of IL-23 mRNA by these cells increased significantly following incubation with ArtinM compared to medium (S2 Fig). Once stimulated with ArtinM, peritoneal macrophages from WT, TLR2 KO and CD14 KO mice increased the IL-23 mRNA expression significantly. Furthermore, the lack of TLR2 or CD14 showed no significant difference in the expression of IL-23 mRNA in the presence of ArtinM (Fig 2C). Taken together, the results reported in this section show that the increased IL-23 expression by ArtinM-stimulated macrophages might constitute an efficient manner of promoting Th17 immunity.

### IL-1R-mediated activation of macrophages contributes to the IL-17 production promoted by ArtinM

**Fig 3:**
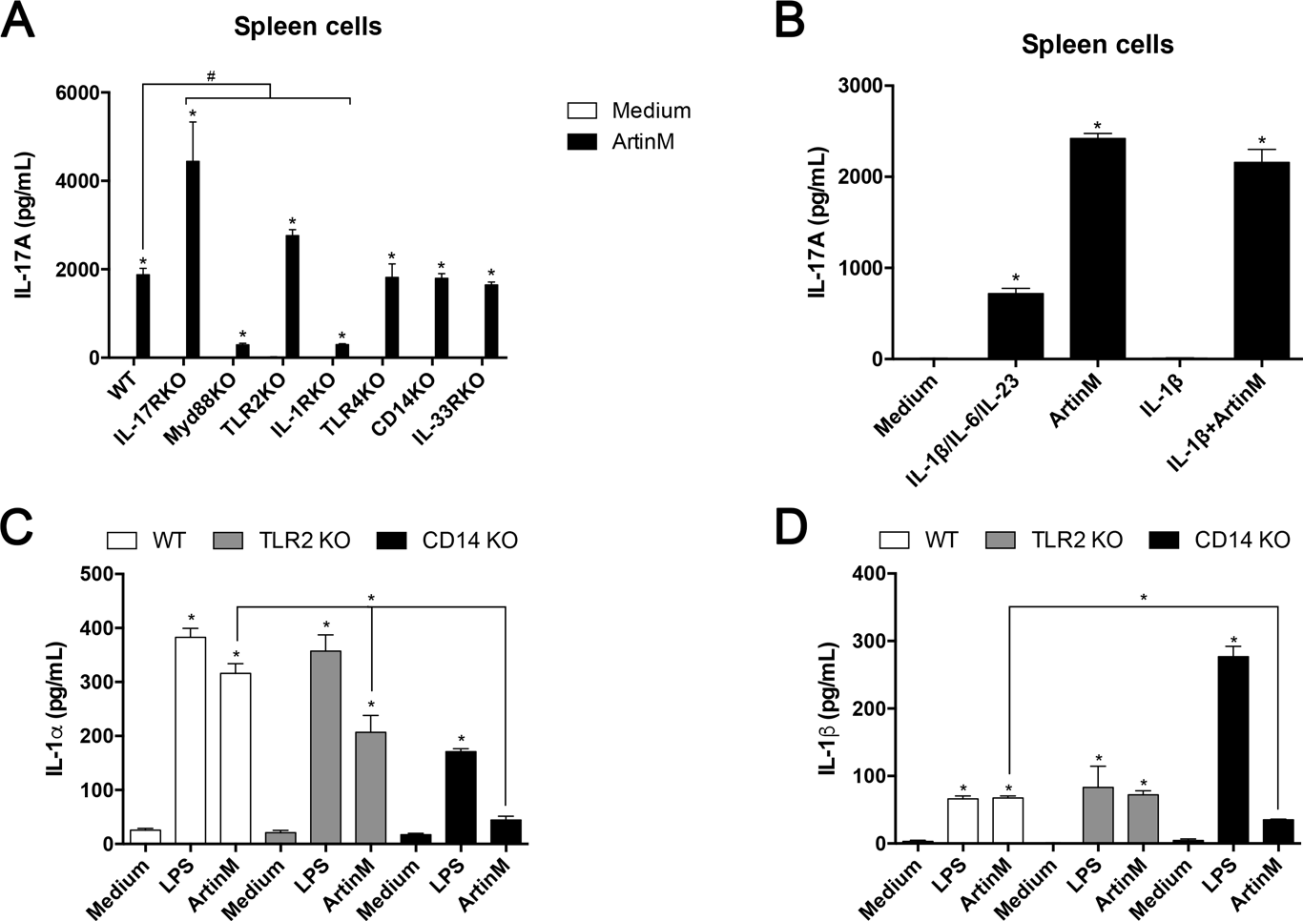
IL-1R signaling contributes to IL-17 production induced by ArtinM. (**A**) Spleen cells (2 × 10^6^/mL) from C57BL/6, IL-17R KO, MyD88 KO, TLR2 KO, IL-1R KO, TLR4 KO, CD14 KO, and IL-33R KO mice were stimulated with ArtinM (1.25 μg/mL) for 48 h at 37°C. PMA plus ionomycin (81 nM + 5 μM) and medium were used as positive and negative controls, respectively. The IL-17 levels in the cell supernatants were determined and compared to that of the unstimulated cells (Medium); the IL-17 levels induced by ArtinM in WT and KO mice were also compared. PMA plus Ionomycin (positive control) induced cells of all mice strains to produce IL-17, a response that was significantly reduced in cells from IL-1R KO mice (not shown). **(B)** Murine spleen cells (2 × 10^6^/mL) from WT mice were stimulated for 48 h with ArtinM (1.25 μg/mL) alone or in association with IL-1β (10 ng/mL). As a control, IL-1β (10 ng/mL) was used alone, and this cytokine was associated with IL-6 (10 ng/mL) and IL-23 (10 ng/mL) as positive control. Medium alone was an additional negative control. (**C and D**) Peritoneal macrophages (2 × 10^6^/mL) obtained from C57BL/6 (white bars), TLR2 KO (gray bars) and CD14 KO (black bars) mice were incubated for 48 h with ArtinM (1.25 μg/mL). LPS (1 μg/mL) and medium alone were used as positive and negative controls, respectively. IL-1α (**C**) and IL-1β (**D**) were determined by ELISA. (**A-D**) Statistical comparisons were established between unstimulated and stimulated cells, and (**A**, **C** and **D**) additional between the cytokines levels induced by ArtinM in WT and KO mice. The results are expressed as mean ± SEM; differences were significant when p < 0.05 (*).

Since macrophages are important for the enhanced IL-17 production by spleen cells stimulated with ArtinM, we investigated whether its known targets on macrophages, TLR2, and CD14, could be involved in the augmentation of IL-17 release. Then, we stimulated spleen cells from TLR2 KO or CD14 KO mice with ArtinM and measured the levels of released IL-17. As shown in Fig 3A, the lack of TLR2 or CD14 did not impair the IL-17 release induced by ArtinM. On the other hand, the ArtinM-stimulated production of IL-17 significantly decreased when we assayed spleen cells from MyD88 KO or IL-1R KO mice (Fig 3A). The addition of exogenous IL-1β (10 ng/mL) exerted no effect on the levels of IL-17 produced by ArtinM-stimulated spleen cells from WT mice (Fig 3B). Nonetheless, it is possible that the addition of higher concentration of exogenous IL-1β could promote the IL-17 production by acting synergistically with ArtinM. Moreover, the IL-1α production by peritoneal macrophages from TLR2 KO and CD14 KO mice decreased significantly compared to WT mice after the incubation with ArtinM (Fig 3C), and the release of IL-1β by peritoneal macrophages was blocked in the absence of CD14 (Fig 3D). The ArtinM-stimulated IL-17 production by spleen cells did not decrease in the absence of TLR-4, IL-17R, or IL-33R (Fig 3A).

**Fig 4:**
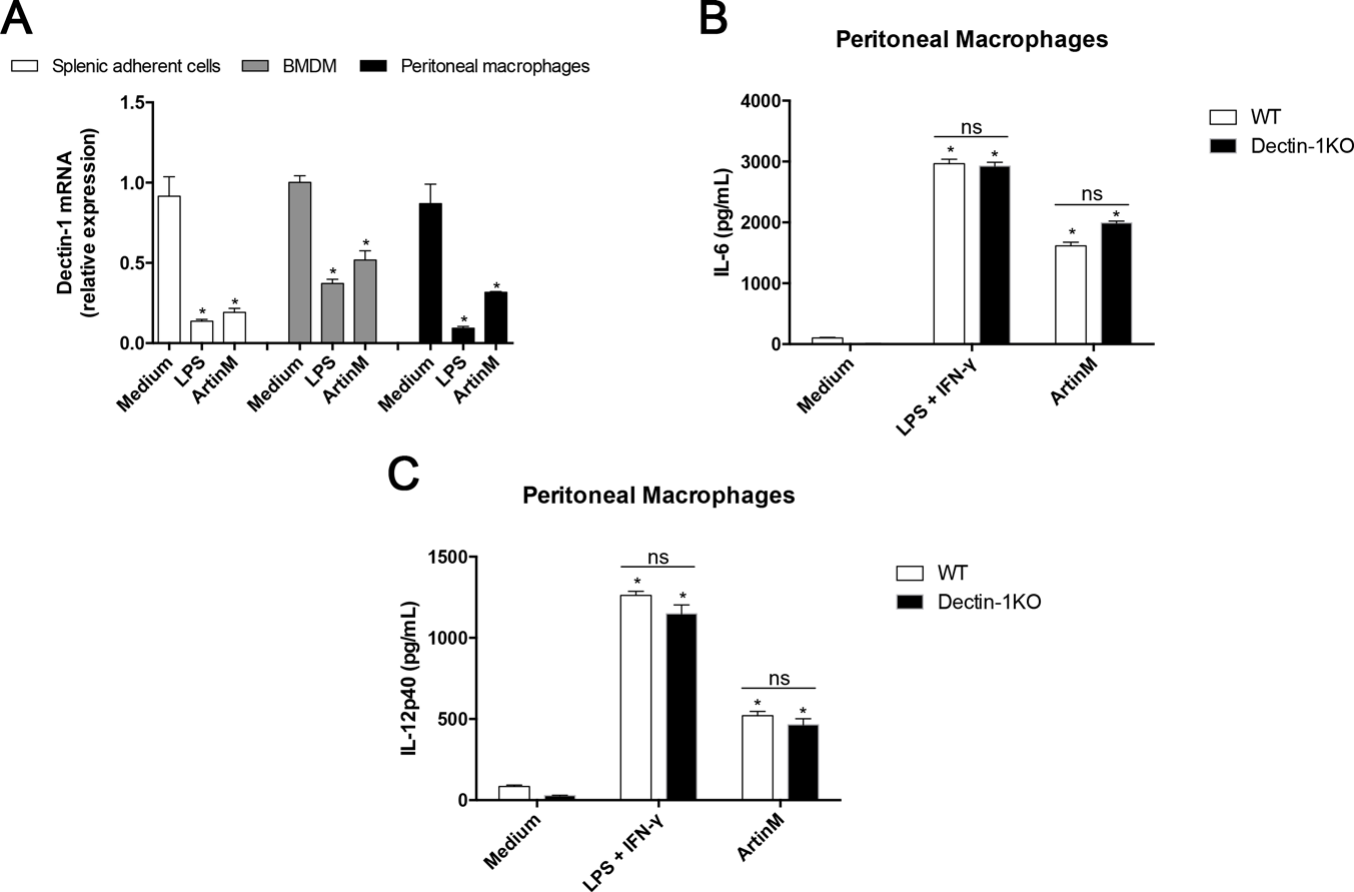
Dectin-1 role in the peritoneal macrophages activation induced by ArtinM. (**A**) Total RNA of splenic adherent cells (2 × 10^6^/mL), BMDMs (1 × 10^6^/mL), and peritoneal macrophages (1 × 10^6^/mL), from C57BL/6 mice that were incubated with ArtinM (1.25 μg/mL; for 7 h at 37°C), was used for real-time quantitative PCR of Dectin-1 mRNA as described in Materials and Methods. Medium and LPS (1 μg/mL) were used as negative and positive controls, respectively. The results are expressed as the relative expression of Dectin-1 normalized to β-actin expression, and compared to medium values. (**B** and **C**) Peritoneal macrophages (1 × 10^6^/mL) obtained from C57BL/6 (white bars) and Dectin-1 KO (black bars) mice were incubated for 48 h with ArtinM (1.25 μg/mL). LPS plus IFN-γ (1 μg/mL + 2 ng/mL) and medium alone were used as positive and negative controls, respectively. The cell supernatants levels of IL-6 (**B**) and IL-12p40 (**C**) were determined by ELISA. (**A-C**) Statistical comparisons were established between unstimulated and stimulated cells and between the IL-17 levels induced by ArtinM in WT and KO mice. The results are expressed as mean ± SEM; differences were significant when p < 0.05 (*) and in other cases were nonsignificant (ns).

It is known that the activation of macrophages by Dectin-1 agonists can drive the IL-17-producing T cells. In addition, Dectin-1 interacts with other MyD88-coupled TLRs resulting in synergistic induction of IL-23. Then, we analyzed the Dectin-1 expression by splenic adherent cells, BMDMs and peritoneal macrophages stimulated with ArtinM and verified that they had reduced Dectin-1 mRNA expression compared to unstimulated cells (Fig 4A). A similar decrease was observed in cells simulated with LPS (positive control). We then investigated whether Dectin-1 could influence the activation of peritoneal macrophages by ArtinM, as manifested by enhanced IL-6 (Fig 4B) and IL-12 (Fig 4C) production. The response to ArtinM, as well as to LPS+IFN-γ (positive control), was preserved in macrophages from Dectin-1 KO mice (Fig 4B and 4C). These findings suggest that ArtinM induces macrophage activation in a way that does not depend on Dectin-1.

### CD4^+^ T cells are directly targeted by ArtinM to induce IL-17 production

**Fig 5:**
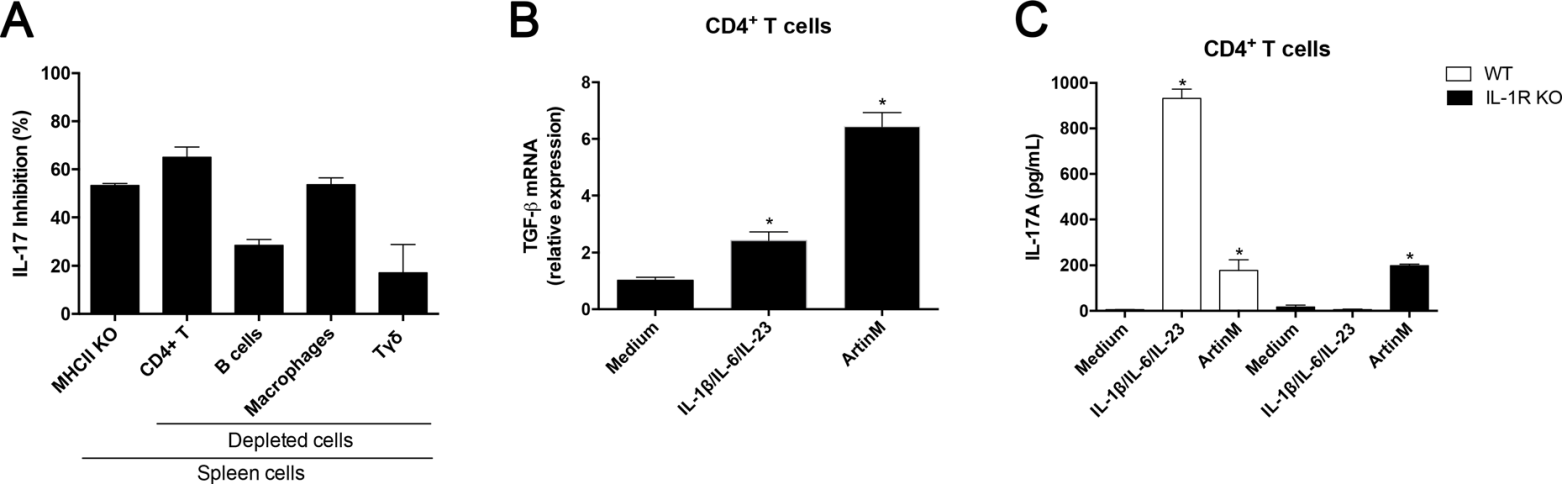
Identification of the spleen cell population accounting for the IL-17 production induced by ArtinM. (**A**) Spleen cells from C57BL/6 mice, depleted with CD4^+^ T-, B-, macrophages, or Tγδ-cells were used; spleen cells from MHC-II KO mice (CD4^+^ T cell-deficient) were additionally assayed. The cell suspensions (2 × 10^6^/mL) were incubated with ArtinM (1.25 μg/mL) at 37°C for 48 h, and the IL-17 levels in the cell supernatants were determined by ELISA. Obtained measurements were compared to that of the non-depleted cells of C57BL/6 mice and relative differences were expressed in percentage of IL-17 inhibition. (**B** and **C**) Isolated CD4^+^ T cells (1 × 10^6^/mL) from spleen cells from C57BL/6 or IL-1R KO mice were incubated at 37°C for 48 h with ArtinM (1.25 μg/mL) or with a mixture of IL-1β, IL-6, and IL-23 (20 ng/mL; 20 ng/mL; 20 ng/mL). Total RNA of CD4^+^ T cells from C57BL/6 mice was used for real-time quantitative PCR of TGF-β mRNA (**B**). Besides, the CD4^+^ T cells supernatants from C57BL/6 and IL-1R KO mice were assessed for IL-17 levels by ELISA (**C**). The results are expressed as mean ± SEM; differences were considered significant when p < 0.05(*) as compared to medium.

Although spleen cells lacking IL-23, MyD88, or IL-1R produced much less IL-17 in response to ArtinM than WT cells, suggesting that IL-17 production resulted from the lectin interaction with innate immune cells, we still should investigate if the occurrence of ArtinM concomitant interaction with cells of the adaptive immunity accounts for the IL-17 response. To elucidate this question, we measured IL-17 production by spleen cell suspensions that were depleted of either of the following cell population: macrophages (CD11b^+^), B lymphocytes (CD19^+^), CD4^+^ T cells (non-CD4^+^ T cells were magnetically adsorbed), or Tγδ cells (TCRγδ). The results obtained after 48 h stimulation with ArtinM were compared to those of the non-depleted spleen cells and were expressed as the percentage of inhibition of IL-17 production. The depletion of macrophages inhibited the IL-17 production by 56%. The lack of B-cells, which constitute the most prevalent cell population in the spleen, caused 30% reduction in IL-17 secretion (Fig 5A). As expected, the depletion of the main IL-17 producers, CD4^+^ T cells, or Tγδ-cells, decreased the IL-17 release, where the maximal inhibition was observed due to the depletion of CD4^+^ T cells (Fig 5A). In the same context, we assayed spleen cells from MHC-II KO mice (CD4^+^ T cell-deficient), which were also associated with the inhibition of IL-17 production in response to ArtinM (Fig 5A). Depletion of CD4^+^ T cells or the use of cells from MHC-II-KO mice inhibited the IL-17 release by 60%.

Considering our previous report that an ArtinM direct interaction with CD4^+^ T cells promotes IL-2 production [25], we hypothesized that the induction of IL-17 release by the lectin could be also attributed to a direct targeting of CD4^+^ T cells. We assessed this question by using CD4^+^ T cells that were isolated from suspensions of mouse spleen cells and incubated with ArtinM (1.25 μg/mL) for 48 h. These isolated cells had augmented expression of TGF-β mRNA compared to the non-stimulated cells (Fig 5B). Moreover, isolated CD4^+^ T cells from WT and IL-1R KO mice under ArtinM stimulus produced high levels of IL-17 than the non-stimulated cells (Fig 5C). Thus, a direct interaction with CD4^+^ T cells contributes to the ArtinM-induced IL-17 production.

### ArtinM interaction with CD3 is essential for the induction of IL-17 production

**Fig 6:**
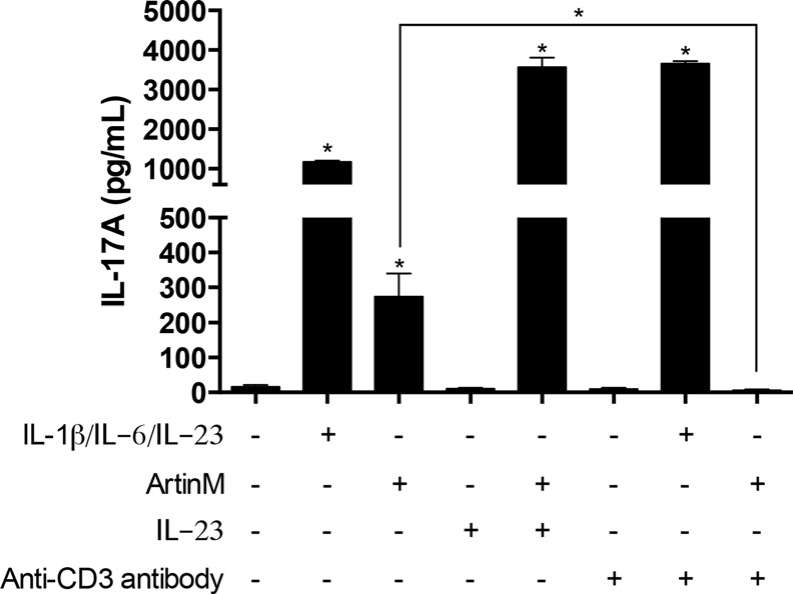
The effect of anti-CD3 antibody in the IL-17 production induced by ArtinM. Isolated CD4^+^ T cells (2 × 10^6^/mL) obtained from IL-23 KO mice were incubated at 37°C for 40 min with the anti-CD3 antibody (15 μg/mL; clone 17A2) or IgG Isotype control (15 μg/mL; A19-3 clone). Cell suspensions were stimulated for 48 h with ArtinM (1.25 μg/mL), with a mixture of IL-1β/IL-6/IL-23 (20 ng/mL; 20 ng/mL; 20 ng/mL), with IL-23 alone (20 ng/mL) or with medium alone. IL-17 level in the cell supernatants was measured by ELISA. The results are expressed as mean ± SEM. The values were compared to unstimulated cells (Medium), or between ArtinM-stimulated cells that were pre-incubated or not with anti-CD3 antibody. Differences were considered significant when p < 0.05 (*).

We had reported that ArtinM depends on the interaction with CD3 to promote the IL-2 production by CD4^+^ T cells [25]; in addition, we verified that anti-CD3 antibody exerted a significant inhibitory effect on the ArtinM-induced IL-17 release by spleen cells (S3 Fig). Considering that a direct recognition of CD4^+^ T cells by ArtinM promotes IL-17 production, we investigated whether CD3 could be the target responsible for this activity. In order to avoid the effect of IL-23 on the IL-17 production, the analysis was performed by using CD4^+^ T cells from IL-23 KO mice, which were isolated using magnetic beads. The cells were pre-incubated with anti-CD3 antibody and, after washing, stimulated with ArtinM. Fig 5 shows that the CD4^+^ T cells that reacted with anti-CD3 antibody reduced the IL-17 production stimulated by ArtinM significantly, whereas a similar procedure had no effect on the IL-17 production induced by exogenous IL-23 in association with IL-1β and IL-6. Furthermore, we found that addition of exogenous IL-23 to CD4^+^ T cells acts synergistically with ArtinM to induce IL-17 production (Fig 6), confirming the observation performed by using spleen cell suspension (Fig 2B). Our results indicate that the ArtinM-induced IL-17 production by CD4^+^ T cells depends on the lectin interaction with CD3.

## Discussion

The known ArtinM interaction with TLR2 N-glycans on innate immune cells is critical for inducing Th1 cytokines production and modulating immunity for protection against infections by intracellular pathogens [21, 22]. Moreover, ArtinM is associated with the induction of Th17 response, an effect that was observed when the lectin was administered to *C*. *albicans-*infected mice [24]. The mechanism underlying the induction of Th1 immunity receives the contribution of the ArtinM effects on CD4^+^ T cells [25]. The evidences that ArtinM has effects on cells of the adaptive immunity are expanded in the present study, with the demonstration that ArtinM induces IL-17 production in CD4^+^ T cells. Also, we showed that ArtinM induces APCs to express IL-23 and to secrete IL-1, which are known to positively influence the IL-17 production. Herein, we also demonstrated that IL-23 acts synergistically with ArtinM on CD4^+^ T cells in promoting IL-17 release. These observations indicated that ArtinM could act on cells of the innate and adaptive immune system for the development of Th17 immunity.

Considering that ArtinM interaction with TLR2 N-glycans induces the production of proinflammatory cytokines [21], we postulated that ArtinM sugar-recognition property also accounts for the spleen cells activation leading to IL-17 production. We verified that ArtinM induces higher IL-17 production in spleen cells from C57BL/6 mice than from BALB/c mice. This finding can be associated with the diversity in glycosylation within populations of the same species [26], because each N-glycan type exhibits a core unit and the extension of this core results in strain-specific molecules [27]. This observation is evidenced in glycoproteins that exhibit a highly heterogeneous glycosylation pattern, as described in the MUP complex (major urinary protein) with the glycan heterogeneity related to the degree of sialylation and the terminal galactose residues [28]. In addition, other factors could influence the IL-17 production induced by ArtinM on spleen cells from C57BL/6 and BALB/c, such as differences in TLRs expression by splenic dendritic cells from BALB/c and C57BL/6 mice [29] and a lower frequency of CD4^+^CD25^+^ Treg cells in secondary lymphoid tissues of C57BL/6 than in those of BALB/c mice [30]. This fact is relevant because CD4^+^CD25^+^ Treg cells may inhibit the development of Th17 cells.

The activity of inducing IL-17 production had been previously reported only for Cramoll and ConA [31]. We verified that several lectins, with distinct sugar-binding specificities, induce IL-17 production by spleen cells and showed that ArtinM promotes the highest release in comparison to ConA, E-PHA, L-PHA, MAL, SNA, Jacalin, and UEA. We also demonstrated that the levels of IL-17 production are independent of the magnitude of binding of each lectin to the cells surface. Indeed the selectivity of binding, which is dictated by the lectin specificity, seems to determine the cell response.

The ArtinM effect on IL-17 production by spleen cell suspensions motivated us to evaluate the dependence of this response on cytokines secreted by cells of both, innate and adaptive immune system. It is known that Th1 and Th2 cytokines are potent inhibitors of Th17 cell differentiation [3, 32]. Consistently, ArtinM stimulated higher IL-17 production by spleen cells obtained from IL-4, IL-10, or IFN-γ KO mice than from WT mice. Meanwhile, the absence of IL-23 and IL-1R decreased the IL-17 release promoted by ArtinM indicating that IL-17 production indeed depends on IL-23 and IL-1 to be sustained at high levels [4]. We verified that macrophages constitute an important source of the IL-23 and IL-1 induced by ArtinM, especially those derived *in vitro* from bone marrow cells.

We described that ArtinM interacts with TLR2 N-glycan(s) [21], furthermore, the stimulation with both TLR2 and Dectin-1 ligands may lead to amplification of the IL-17 production [13]. However, we showed that ArtinM does not depend on Dectin-1 to activate macrophages. Concerning the IL-17 production, the ArtinM interaction with TLR2 is not crucial, since similar levels of IL-17 were released by TLR2 KO cells or WT cells. Recently, we verified that CD14 N-glycan(s) are targeted by ArtinM, establishing an interaction with APCs that is critical for the IL-12 production (unpublished data). Herein, the absence of CD14 did not impair the IL-17 production induced by ArtinM. These facts motivated us to evaluate the involvement of the adaptor molecule MyD88, which participates in cell signaling triggered by members of TLR and IL-1R families and provides a link between these pathways [8]. In addition, it is known that IL-1 contributes to Th17 cell generation [7]. Coherently, the ArtinM-stimulated release of IL-17 was strongly inhibited when spleen cells from MyD88 KO or IL-1R KO mice were assayed, supporting the idea that IL-1 signaling constitutes a mechanism through which ArtinM induces IL-17 production. Moreover, we demonstrated that ArtinM promotes the IL-1 production by macrophages through recognition of N-linked glycans of the CD14.

We showed that IL-23 acts synergistically with ArtinM to induce IL-17 production by spleen cells as well as by purified CD4^+^ T cells. We also verified that the levels of IL-17 production in response to ArtinM were inhibited, but not blocked, when the assayed spleen cell suspensions were depleted of macrophages or B cells, whereas Tγδ-cells, which comprise 1–5% of the total number of lymphocytes in mice [33] accounted for about 20% of the IL-17 production stimulated by ArtinM. Considering the IL-17 production that persisted in spite of cell depletion procedures, or in the absence of several mediators, we hypothesized that a direct interaction of ArtinM with CD4^+^ T cells could account for IL-17 release. This idea was reinforced by our previous finding that the IL-2 production by murine CD4^+^ T cells induced by ArtinM results from the lectin interaction with CD3 on the cell surface [25]. Herein, we found that the IL-17 production stimulated by ArtinM is prominently attributed to CD4^+^ T cells, with CD3 being the ArtinM glycotarget responsible for triggering IL-17 release. The relevance of the ArtinM interaction with CD3 for the induction of IL-17 was demonstrated by a functional competition assay between ArtinM and anti-CD3 antibodies, in which the specific antibodies blocked the ArtinM effect of inducing IL-17 on CD4^+^ T cells. Thus, we conclude that ArtinM interaction with CD3 is a direct mechanism through which ArtinM induces IL-17 production by CD4^+^ T cells.

In conclusion, ArtinM stimulates the production of IL-17 by CD4+ T cells in two major ways. The first mechanism involves the ArtinM activity of inducing APCs to produce IL-23 and IL-1, whose effect in maintaining high levels of IL-17 release is well known. An additional mechanism concerns the ArtinM property of recognizing CD3 N-glycans; this direct interaction established on CD4^+^ T cells triggered IL-17 production in the lack of IL-23 and IL-1. The present study allows an understanding of the origin of the Th17 immunity induced *in vivo* by ArtinM administration and contributes to defining new strategies to modulate immunity through carbohydrate recognition.

## Supporting Information

S1 Fig IL-17 production by spleen cells stimulated by variable concentrations of ArtinM. Murine spleen cells (2 × 10^6^/mL) from C57BL/6 and BALB/c mice were incubated at 37°C for 12, 24, and 48 h under stimulus of various concentrations of PMA plus ionomycin (**A**), LPS plus P3C4 (**A**) or ArtinM (**B**), as indicated in the figure. Medium alone was used as negative control. IL-17 levels in culture supernatants were measured by ELISA. The results are expressed as mean ± SEM, with significant differences when p < 0.05(*) compared to unstimulated cells.
(TIF)

S2 Fig IL-23 expression in splenic adherent cells and BMDM in response to ArtinM stimulus. Splenic adherent cells (2 × 10^6^/mL) and BMDM (1 × 10^6^/mL) from C57BL/6 mice were incubated with ArtinM (1.25 μg/mL) for 7 h and then the extracted RNA was used for real-time quantitative PCR of IL-23 mRNA, as described in Materials and Methods. Medium and LPS (1 μg/mL) were used as negative and positive controls, respectively. The results are expressed as the relative expression of IL-23 normalized to β-actin expression. The results are expressed as mean ± SEM, and the expression of IL-23 were compared to that of the unstimulated cells (Medium).
(TIF)

S3 Fig Pre-incubation with anti-CD3 antibody: effect in the spleen cell response to ArtinM stimulus. Spleen cells (2 × 10^6^/mL) from C57BL/6 mice were pre-incubated with the anti-CD3 antibody (15 μg/mL; clone 17A2) or IgG Isotype control (15 μg/mL; A19-3 clone), as indicated in the figure. After washing, the cells were incubated at 37°C for 40 min with ArtinM (1.25 μg/mL). A mixture of IL-1β/IL-6/IL-23 (20 ng/mL; 20 ng/mL; 20 ng/mL) or medium alone was used as positive and negative controls, respectively. ELISA was used to measure the IL-17 production levels in the cell supernatants. The results are expressed as mean ± SEM. The values were compared to those of the unstimulated cells, and additional comparison was established between ArtinM-stimulated cells that were pre-incubated or not with anti-CD3 antibody. Differences were considered significant when p < 0.05 (*).
(TIF)
