# Supplementary figures and images for: IL-17 Induction by ArtinM is Due to Stimulation of IL-23 and IL-1 Release and/or Interaction with CD3 in CD4+ T Cells
Source: PLoS One. 2016 Feb 22;11(2):e0149721. doi: 10.1371/journal.pone.0149721 (PMC4767177; doi:10.1371/journal.pone.0149721)

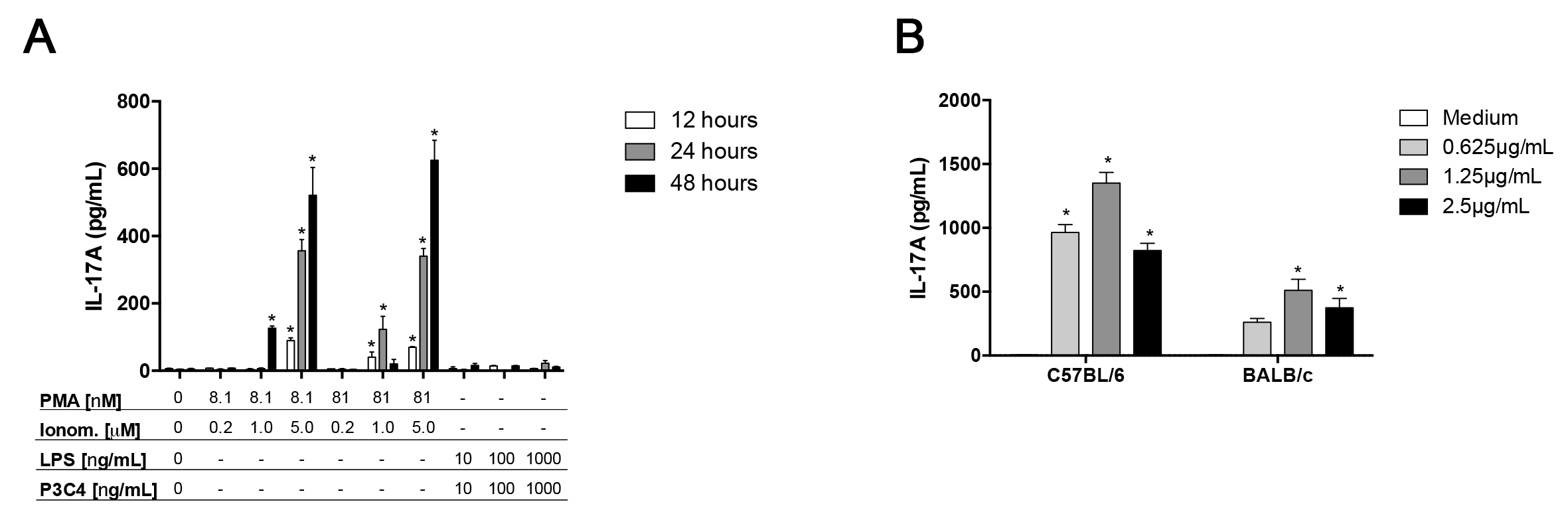

Supplement: S1 Fig — Murine spleen cells (2 × 10^6^/mL) from C57BL/6 and BALB/c mice were incubated at 37°C for 12, 24, and 48 h under stimulus of various concentrations of PMA plus ionomycin (A), LPS plus P3C4 (A) or ArtinM (B), as indicated in the figure. Medium alone was used as negative control. IL-17 levels in culture supernatants were measured by ELISA. The results are expressed as mean ± SEM, with significant differences when p < 0.05(*) compared to unstimulated cells. (TIF) [file pone.0149721.s001.tif]

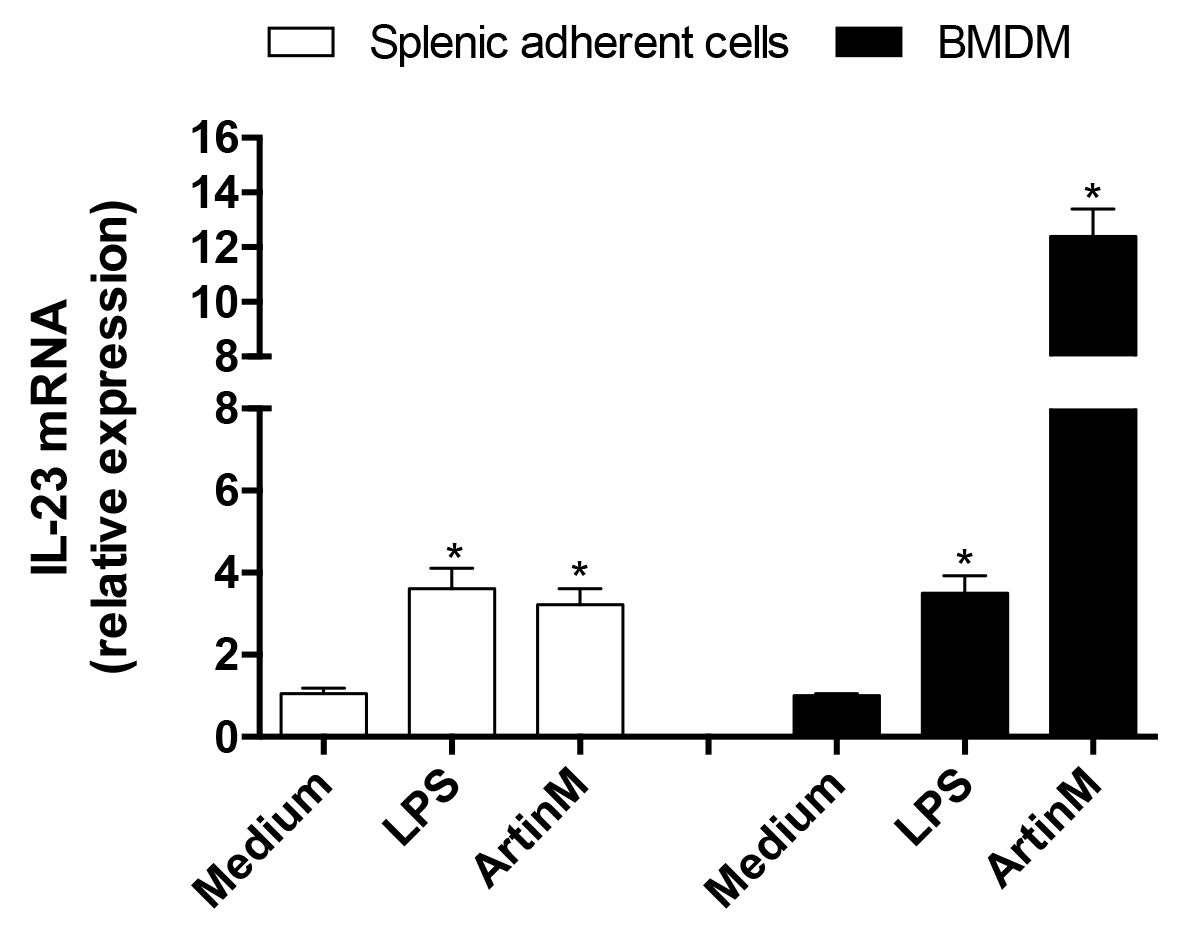

Supplement: S2 Fig — Splenic adherent cells (2 × 10^6^/mL) and BMDM (1 × 10^6^/mL) from C57BL/6 mice were incubated with ArtinM (1.25 μg/mL) for 7 h and then the extracted RNA was used for real-time quantitative PCR of IL-23 mRNA, as described in Materials and Methods. Medium and LPS (1 μg/mL) were used as negative and positive controls, respectively. The results are expressed as the relative expression of IL-23 normalized to β-actin expression. The results are expressed as mean ± SEM, and the expression of IL-23 were compared to that of the unstimulated cells (Medium). (TIF) [file pone.0149721.s002.tif]

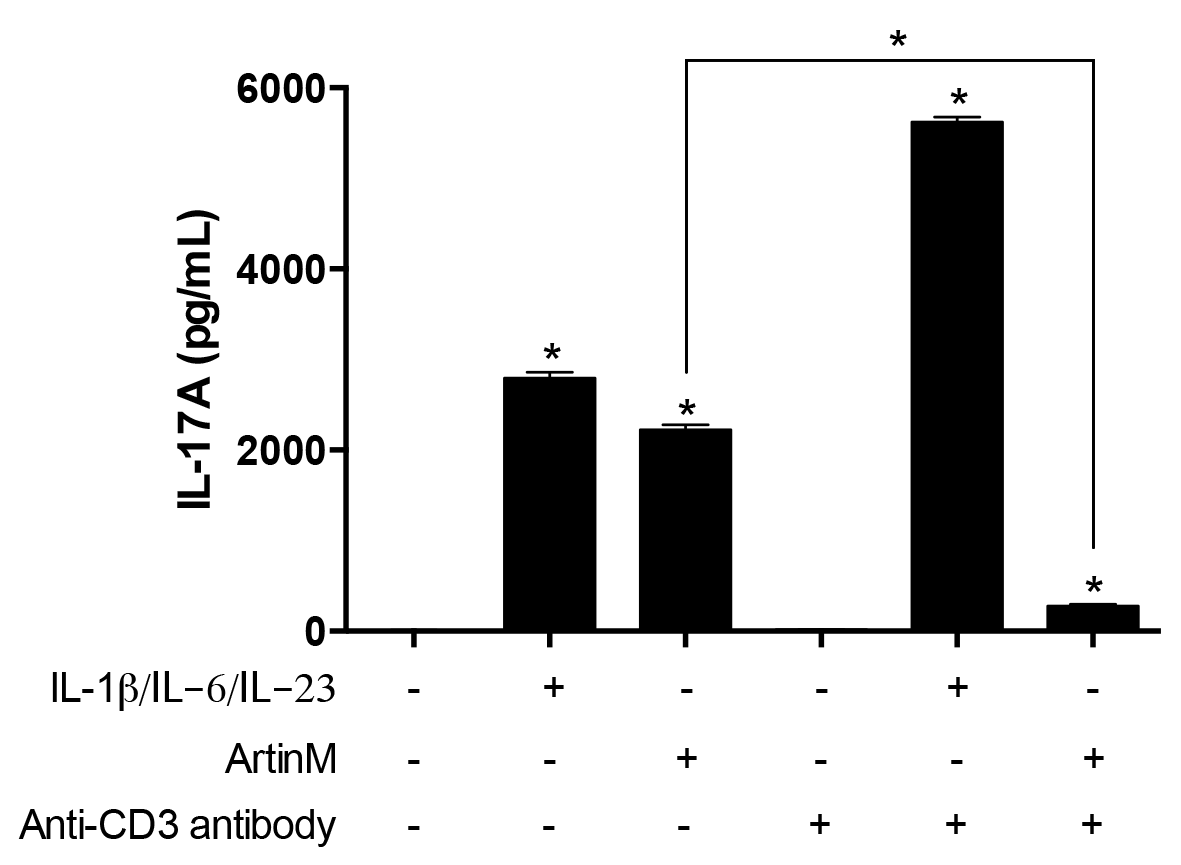

Supplement: S3 Fig — Spleen cells (2 × 10^6^/mL) from C57BL/6 mice were pre-incubated with the anti-CD3 antibody (15 μg/mL; clone 17A2) or IgG Isotype control (15 μg/mL; A19-3 clone), as indicated in the figure. After washing, the cells were incubated at 37°C for 40 min with ArtinM (1.25 μg/mL). A mixture of IL-1β/IL-6/IL-23 (20 ng/mL; 20 ng/mL; 20 ng/mL) or medium alone was used as positive and negative controls, respectively. ELISA was used to measure the IL-17 production levels in the cell supernatants. The results are expressed as mean ± SEM. The values were compared to those of the unstimulated cells, and additional comparison was established between ArtinM-stimulated cells that were pre-incubated or not with anti-CD3 antibody. Differences were considered significant when p < 0.05 (*). (TIF) [file pone.0149721.s003.tif]
